# Supplementary material for: Optimization of differentiation and transcriptomic profile of THP-1 cells into macrophage by PMA
Source: PLoS One. 2023 Jul 17;18(7):e0286056. doi: 10.1371/journal.pone.0286056 (PMC10351730; doi:10.1371/journal.pone.0286056)
Supplement: S1 File — (ZIP) [file pone.0286056.s001.zip › supporting files/RNA-seq report.zip!/RNA-seq report/4_GOEnrichment_result/induce_vs_control/DAG/induce_vs_control.DEG_up_bp_DAG.pdf]

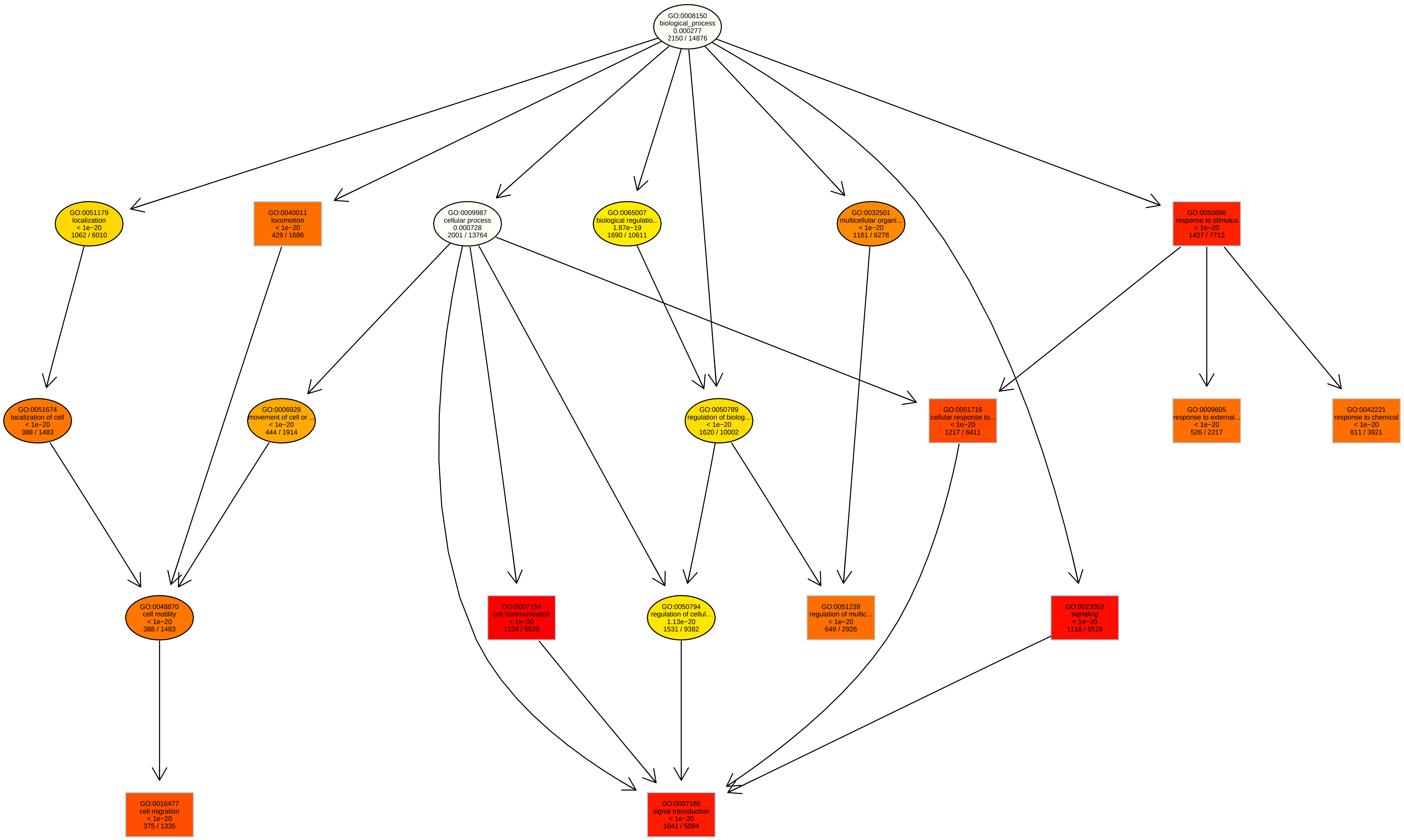

GO:0008150 biological_process 0.000277 2150 / 14876
GO:0051179 localization < 1e−20 1062 / 6010
GO:0040011 locomotion < 1e−20 429 / 1686
GO:0009987 cellular process 0.000728 2001 / 13764
GO:0065007 biological regulatio... 1.87e−19 1690 / 10611
GO:0032501 multicellular organi... < 1e−20 1161 / 6278
GO:0050896 response to stimulus < 1e−20 1427 / 7713
GO:0051674 localization of cell < 1e−20 388 / 1483
GO:0006928 movement of cell or ... < 1e−20 444 / 1914
GO:0050789 regulation of biolog... < 1e−20 1620 / 10002
GO:0051716 cellular response to... < 1e−20 1217 / 6411
GO:0009605 response to external... < 1e−20 526 / 2217
GO:0042221 response to chemical < 1e−20 811 / 3921
GO:0048870 cell motility < 1e−20 388 / 1483
GO:0007154 cell communication < 1e−20 1124 / 5539
GO:0050794 regulation of cellul... 1.13e−20 1531 / 9382
GO:0051239 regulation of multic... < 1e−20 649 / 2926
GO:0023052 signaling < 1e−20 1116 / 5516
GO:0016477 cell migration < 1e−20 375 / 1335
GO:0007165 signal transduction < 1e−20 1041 / 5094
